# Characterization of immune microenvironment in patients with HPV-positive and negative head and neck cancer

**DOI:** 10.1038/s41597-023-02611-3

**Published:** 2023-10-12

**Authors:** Zhongqiu Wang, Qingxin Wang, Yuxuan Tao, Jingru Chen, Zhiyong Yuan, Peiguo Wang

**Affiliations:** 1 Department of Radiation Oncology, Tianjin Medical University Cancer Institute & Hospital, National Clinical Research Center for Cancer, Tianjin’s Clinical Research Center for Cancer, Key Laboratory of Cancer Prevention and Therapy, West Huanhu Road, West River District, Tianjin, 300060 China; 2 School of Precision Instrument and Opto-electronics Engineering, Tianjin University, 92 Weijin Road, Nankai District, Tianjin, 300072 China

**Keywords:** Head and neck cancer, Cancer microenvironment

## Abstract

Human papillomavirus (HPV) status strongly predicts positive clinical outcomes in patients with head and neck squamous cell cancer (HNSCC); however, the potential reasons have not been fully elucidated. Here, we characterized the immune context in HPV+ and HPV− HNSCC by integrating scRNA-seq and bulk RNA-seq data. In scRNA-seq data, HPV + HNSCC displayed increased B cells, plasma cells, CD4^+^ effector T cells, and decreased macrophages and mast cells. This finding was validated using bulk-cell data. Plasma cells predicted improved survival, and macrophages were associated with survival disadvantage. 1403 upregulated and 1877 downregulated differential expressed genes (DEGs) were obtained. Gene Ontology and KEGG enrichment analysis showed these DEGs focused on cytokine-related activity. Transcriptional analysis of B and plasma cells revealed associations between B-cell surface marker FCER2 and improved survival. *In vitro* assays confirmed the ability of FCER2 to suppress cellular proliferation and migration of HPV + tumors. In conclusion, our analysis revealed a heterogeneous tumor immune environment (TME) for HPV+ and HPV− HNSCC. Further, FCER2^+^ B cells contribute to antitumor immunity.

## Introduction

Head and neck squamous cell cancer (HNSCC) is the eighth most common cancer worldwide, with approximately 750,000 new cases in 2020. Unfortunately, most patients present with locally advanced disease^1^. HNSCC is mainly caused by carcinogens, including excessive consumption of alcohol or tobacco products. HPV is a major risk factor for HNSCC, especially oropharyngeal cancer. The incidence of HPV+ HNSCC has risen substantially, with over half of the cases in the United States testing positive for the HPV genotype (mostly HPV-16)^2,3^. HPV status is a strong predictor of positive clinical outcomes. Past cohort analyses revealed a significant correlation between HPV p16 protein expression and longer overall (OS) and recurrence-free survival (RFS)^4–6^. Despite this, the potential reasons and mechanisms controlling these effects are poorly elucidated.

Immunotherapy has improved survival for patients with HNSCC, demonstrating the efficacy of targeting the immune system and tumor microenvironment (TME). A phase II multi-institutional clinical trial enrolled patients with recurrent and/or metastatic HNSCC treated with cetuximab and nivolumab. The p16-immunostaining was associated with a higher response rate (RR) but did not impact survival. In contrast, in p16+ patients, lower copies of tumor-tissue-HPV DNA were associated with higher RR and longer OS^7^. A patient’s HPV status may underscore important differences in the TME and act as a tumor-specific target for antitumor immunity between HPV+ and HPV− types of HNSCC^8^. For example, HPV + HNSCC tumors are considered “hot,” with additional intratumoral PD-L1^+^ CD68^+^ macrophages and CD3^+^CD8^+^ T cells^9^.

We examined the various immune characteristics of HPV+ and HPV− (represented as p16+ and p16−) HNSCC by integrating one single-cell RNA sequencing dataset (scRNA-seq) and one bulk RNA sequencing dataset from the Gene Expression Omnibus (GEO) database. We identified cytokine-associated features between these two types and a potential regulatory mechanism for HPV + HNSCC. These findings reveal the immune heterogeneity of HNSCC at the single- and bulk-cell levels. *In vitro* experiments were used to validate our results.

## Results

### Immune cell heterogeneity between HPV+ and HPV− HNSCC revealed by single-cell data

**Fig. 1:**
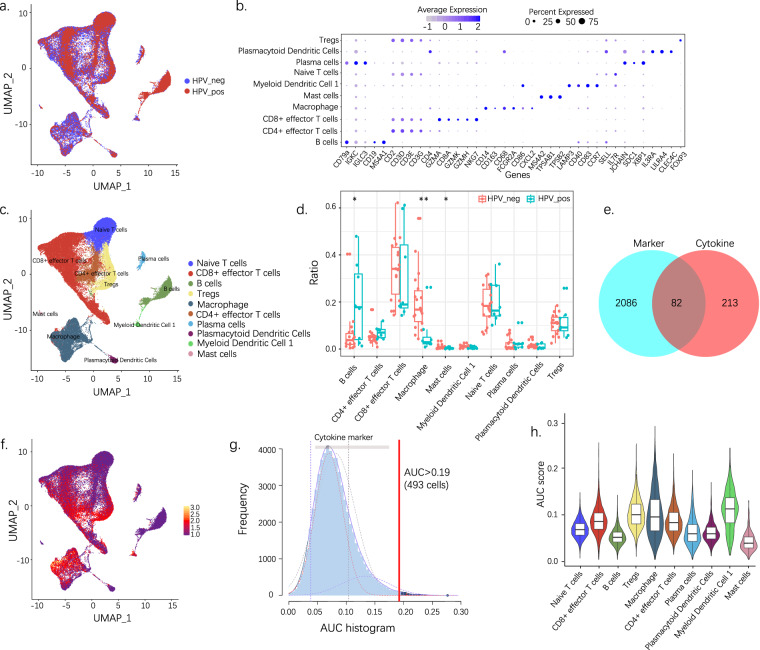
Single-cell analysis reveals the immune cell heterogeneity and cytokine activity between HPV+ and HPV− HNSCC. (**a**) UMAP visualization of different HPV statuses. (**b**) Expression of marker genes for 10 distinct cell types. (**c**) UMAP visualization of predicted cell types. (**d**) Ration comparison of each cell type in HPV+ and HPV− samples. (**e**) Eighty-two differentially expressed cytokines are selected for further analysis. (**f**) The scores of the 82 DEGs. The threshold was chosen as 0.19, and the DEG score of 493 cells exceeded the value. (**g**) UMAP plots based on the DEG score for each cell. Cell clusters with a high score are highlighted. (**h**) Violin plot showing the cytokine activity of each cell type. UMAP, Uniform manifold approximation and projection; DEG, differentially expressed gene. *p < 0.05; **p < 0.01.

We projected scRNA-seq data into a two-dimensional space using uniform manifold approximation and different HPV statuses, as depicted in Fig. 1a. Cluster classification analysis was performed; 10 separate clusters were identified and subsequently assigned to known cell lineages using marker genes (Fig. 1b and Supplementary Fig. 1), as naïve T cells, CD8^+^ effector T cells, B cells, Tregs, macrophages, CD4^+^ effector T cells, plasma cells, plasmacytoid dendritic cells, myeloid dendritic cells 1, and mast cells (Fig. 1c). We then noted the abundance of various immune cell types between the HPV+ and HPV− samples (Fig. 1d). Compared to HPV− HNSCC, the HPV + variety displayed more B cells, plasma cells, CD4^+^ effector T cells, and fewer macrophages and mast cells.

### Cytokine activity of HPV+ and HPV− HNSCC revealed by single-cell data

To investigate the cytokine characteristics of HPV+ and HPV− samples, each cluster’s differentially expressed genes (DEGs) were intersected with the hsa04060 pathway (cytokine–cytokine receptor interaction). We used the AUCell package to calculate each cell’s cytokine activity and estimate the proportion of genes in the gene set that are highly expressed in each cell. Cells expressing many genes from the gene set exhibited higher area under the curve (AUC) values than cells with fewer genes^10^ (Fig. 1e,f). We identified a single AUC peak value. Meanwhile, 493 cells showed relatively higher AUC values when the threshold was set at 0.19 (Fig. 1g). These cells mainly existed in clusters and included Tregs, macrophages, and myeloid dendritic cells 1 (Fig. 1h).

### Immune cell heterogeneity and prognosis between HPV+ and HPV− HNSCC revealed by bulk-cell data

**Fig. 2:**
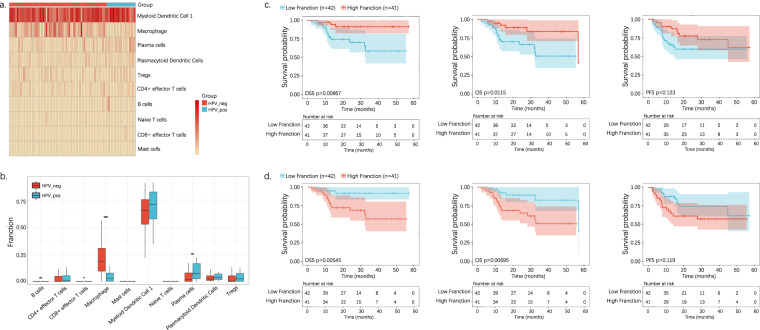
Bulk-cell analysis reveals the immune cell heterogeneity and prognosis between HPV+ and HPV− HNSCC. (**a**) Heatmap showing each cell type in HPV+ and HPV− tumors. (**b**) Ration comparison of each cell type in HPV+ and HPV− tumors. (**c**) Kaplan–Meier analysis of HPV+ and HPV− group dichotomized by the median level of plasma cells. (**d**). Kaplan–Meier analysis of the HPV+ and HPV− groups dichotomized by the median macrophage levels.

We explored gene expression and cytokine-related signaling pathway differences using raw bulk RNA-seq data from the Cancer Genome Atlas (TCGA)-HNSCC cohort. A notable relationship was observed between the HPV+ and HPV− tumors. Macrophages (p < 0.0001) were associated with HPV− tumors; B− and plasma cells (p < 0.05) were mainly enriched in HPV + tumors (Fig. 2a,b).

We next calculated the impact of positive versus negative HPV expression on patient survival by Kaplan–Meier analysis. Patients with higher plasma cell levels demonstrated markedly improved survival, including disease-specific survival (DSS), OS, and progression-free survival (PFS) (Fig. 2c). Otherwise, a significant survival advantage was shown in patients with fewer macrophages (Fig. 2d).

### DEG and functional analysis of HPV+ and HPV− HNSCC screening from bulk-cell data

**Fig. 3:**
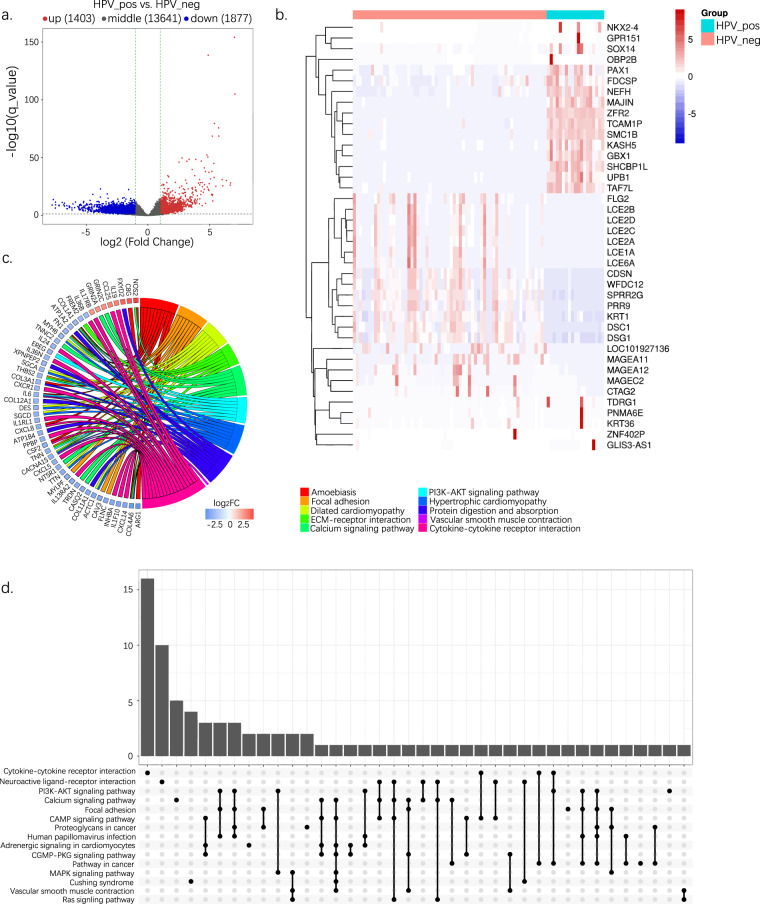
Bulk-cell analysis reveals differentially expressed genes and the functional analysis between HPV+ and HPV− HNSCC. (**a**) Volcano plots showing DEGs in HPV+ and HPV− tumors. (**b**) Expression heatmap for the top 20 significant DEGs. (**c**) GO-biological process enrichment analysis of DEGs. (**d**) KEGG enrichment analysis of DEGs. DEG, differentially expressed gene; GO, Gene Ontology.

Volcano plots were used to compare differences in gene expression (Fig. 3a). A total of 1403 upregulated and 1877 downregulated genes were retained (Supplementary Fig. 2). The top 20 significant DEGs are shown in Fig. 3b. The Gene Ontology (GO) Biological Process enrichment and KEGG enrichment analysis were performed to explore the biological functions of these genes. As shown in Fig. 3c,d, and Supplementary Fig. 3, they were mainly enriched during cytokine–cytokine receptor interactions, such as the CC subfamily-CCR pathway, CXC subfamily-CXCR pathway, TNF family-receptor pathway, and other signaling pathways. Moreover, a list of transcription factors (TFs) was obtained from the database to investigate the transcription-regulated activity of these DEGs, including ChEA, ENCODE, and hTFtarget. Ultimately, 342 TFs were retained (Supplementary Table 1).

### DEG and functional analysis of B cells and plasma cells screening from bulk-cell data

**Fig. 4:**
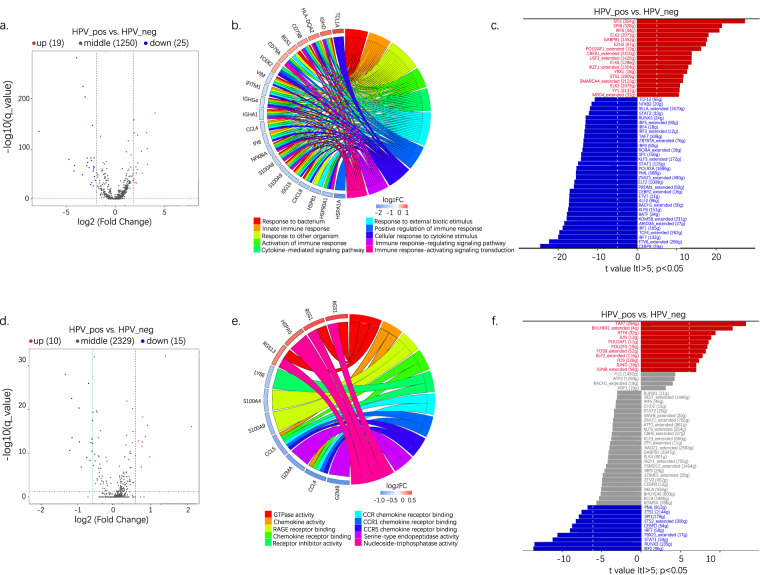
Bulk-cell analysis reveals differentially expressed genes and a functional B and plasma cells analysis. (**a**) Volcano plots show the DEGs of B cells. (**b**) GO enrichment analysis of DEGs of B cells. (**c**) Expression of cytokine-related TFs in B cells. (**d**) Volcano plots showing DEGs of plasma cells. (**e**) GO enrichment analysis of DEGs of plasma cells. (**f**) Expression of cytokine-related TFs in plasma cells. DEG, differentially expressed gene; TF, transcription factor.

As B and plasma cells were found mainly enriched in HPV+ tumors, we further analyzed the signatures of these two clusters. Volcano plots showed that, in HPV+ tumors, 19 B-cell marker genes were upregulated, and 25 genes were downregulated (Fig. 4a). Meanwhile, 10 marker genes were more abundant for plasma cells, and 15 genes were less abundant (Fig. 4d). We identified striking signaling pathway differences during the GO enrichment analysis. For B cells, response to external stimulus, cytokine-mediated signaling pathway, innate immune response, and immune response regulation were enriched in HPV+ tumors (Fig. 4b). The enriched pathway in HPV+ tumors for plasma cells included chemokine activity, chemokine receptor binding, receptor inhibitor activity, CCR chemokine receptor binding, etc. (Fig. 4e). Moreover, transcriptomic studies revealed that, for B cells, 17 TFs—including SPI1, SPIB, IRF8, ELK3, GABPB1, EZH2, POU2AF1_extented, MYBL2_extented, CREB1_extended, USF2_ extended, ELK4, IFZF1_ extended, YBX1, ETS1, SMARCA4_ extended, YY1 and MBD4_ extended—were upregulated in HPV+ tumors vs. in HPV− tumors; and 33 TFs—including TCF1, NF-κB2, RELA_ extended, STAT2 and RUNX3, etc—were downregulated (Fig. 4c). For plasma cells, 11 TFs—including TAF7, BHLHE41_ extended, ATF4, JUN, and POU2AF1, etc—were upregulated in HPV+ tumors. Ten TFs—including PML, ETS1, SPI1, ETS2_ extended, and CEBPD—were downregulated (Fig. 4f).

### B cells expressed marker FCER2 was associated with improved survival

**Fig. 5:**
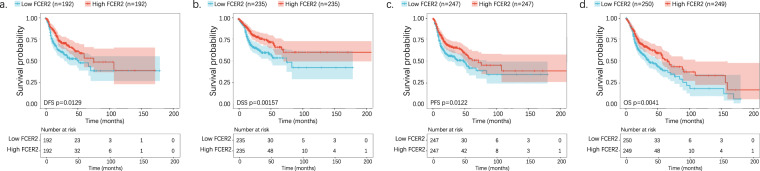
Bulk-cell analysis reveals a correlation between FCER2 expression and prognosis. (**a**) Kaplan–Meier survival curve of DFS between the high- and low-level groups stratified by the FCER2. (**b**) Kaplan–Meier survival curve of DSS between the high- and low-level groups stratified by the FCER2. (**c**) Kaplan–Meier survival curve of PFS between the high- and low-level groups stratified by the FCER2. (**d**) Kaplan–Meier survival curve of OS between the high- and low-level groups stratified by the FCER2. DSF, disease-free survival; DSS, disease-specific survival; PFS, progression-free survival; OS, overall survival.

As indicated by KEGG analysis from bulk and single-cell data, the differentially enriched pathways were intersected to obtain functional DEGs in target clusters. A list of 10 and 5 DEGs was obtained for the B and plasma cell subsets, respectively. Among these DEGs, FCER2—a B-cell surface marker—predicted survival. Higher expression of FCER2 was significantly associated with better survival and resulted in longer disease-free survival (DFS; p = 0.0129, Fig. 5a), DSS (p = 0.00157, Fig. 5b), PFS (p = 0.0122, Fig. 5c), and OS (p = 0.0041, Fig. 5d).

### FCER2^+^ B cells inhibited the proliferation and migration ability of HPV + tumors *in vitro*

**Fig. 6:**
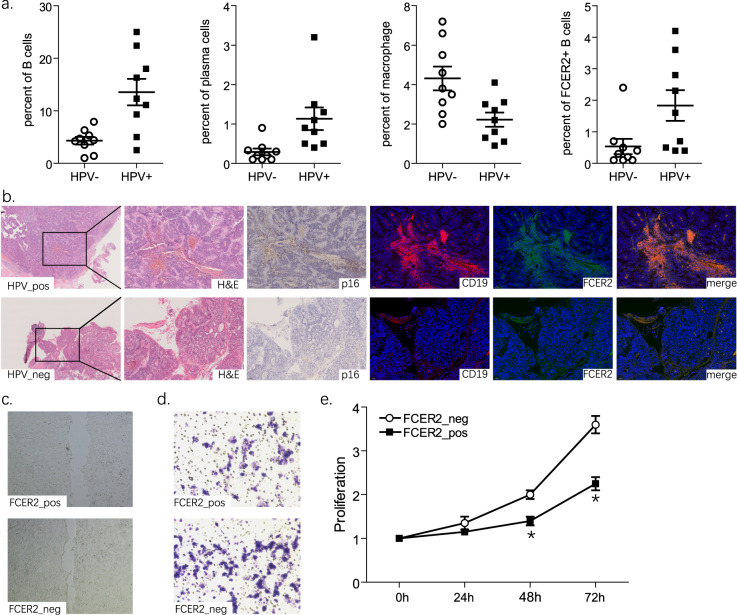
The *in vitro* study reveals that FCER2^+^ B cells inhibit the proliferation and migration ability of HPV+ tumors. (**a**) Flow cytometry analysis of B cells, plasma cells, macrophages, and FCER2^+^ B cells from PBMCs of patients with HNSCC. (**b**) Representative histopathological staining and HPV-p16, as well as FCER2 immunofluorescent staining images of HNSCC samples. FCER2^+^ B cells are cocultured with HPV+ SCC090 cells. (**c**) Scratch experiment showing cell migration. (**d**) Transwell chamber invasion assay showing cell invasion. (**e**) CCK-8 experiment showing cell proliferation. Figure 6 represents three experiments that achieved similar results. *p < 0.05.

To validate the scRNA-seq and bulk RNA-seq data above, additional *in vitro* experiments were performed using nine HPV+ and nine HPV− HNSCC samples. We analyzed the frequency of indicated immune cells in peripheral blood mononuclear cells (PBMCs) with flow cytometry. Compared with patients with HPV− HNSCC, patients with HPV + HNSCC had significantly more B and plasma cells and fewer macrophages (p < 0.05, Fig. 6a). FCER2^+^ B-cell levels were frequently upregulated in HPV+ samples (p < 0.05, Fig. 6a, right side).

Next, we used histopathologic staining to characterize the spatial distribution of FCER2^+^ B cells. We observed an increased number of FCER2^+^ B cells in p16+ samples, predominately in the stroma. However, p16− samples featured fewer FCER2^+^ B cells (Fig. 6b). To investigate their biological functions, we performed proliferation and migration assays after coculturing HPV+ HNSCC and B cells. Upon stimulation with supernatant from a coculture system with FCER2^+^ B cells, the closure rate of SCC090 cells 36 h after the scratch was significantly lower compared to the FCER2^−^ B-cell system (p < 0.05, Fig. 6c). SCC090 cells were placed in the upper transwell system chamber; the lower chamber was filled with B cells. After 24 h, in a system with FCER2^+^ B cells, the average number of SCC090 cells that migrated and adhered to the undersurface was less than with FCER2^−^ B cells (p < 0.05, Fig. 6d). The CCK-8 assay consistently showed a significant decrease in SCC090 cells upon coculturing with FCER2^+^ B cells. These findings suggest reduced proliferation (p < 0.05, Fig. 6e). FCER2^+^ B cells may help inhibit HNSCC cell proliferation and migration *in vitro*.

## Discussion

The TME plays an important role in treatment response and survival. A tumor’s immune subtype signature may predict disease outcomes instead of relying solely on features specific to cancer types^11^. Patients with HPV+ HNSCC tumors enjoy a more favorable prognosis, and not all patients respond well to immunotherapy. Thus, understanding differences in the TME between HPV+ and HPV− tumors may help optimize treatment.

RNA sequencing can provide comprehensive analysis of targeted mRNA transcripts and the complete transcriptome of a particular tissue, i.e. the standard bulk RNA-seq provides an average expression level of gens in different cell types, and the scRNA-seq represents the distribution of gene expression in each subpopulation of cells^12^. In this study, we used datasets including scRNA-seq and bulk-seq, in combination with approaches including flow cytometry, histopathologic staining, and cell models to characterize the TME between HPV+ and HPV− tumors. HPV + samples had more B and plasma cells but decreased macrophage infiltration. Importantly, these differences were associated with improved prognosis. DEG markers of these cell types were enriched in the cytokine-related pathway. Moreover, infiltration of FCER2^+^ B cells contributed to antitumor immunity.

B cells develop in the bone marrow and generate plasma cells in response to T-cell–independent antigens. B and plasma cell functions are poorly understood; however, they likely help control tumor growth in the TME. Notable tumor-infiltrating B cells (sometimes combined with plasma cells) have been identified in breast cancer, ovarian cancer, hepatocellular cancer (HCC), melanoma, nonsmall cell lung cancer (NSCLC), muscle-invasive bladder cancer, colorectal cancer, and pancreatic cancer^12^ despite the heterogenicity of these conditions.

In lung cancer, B cells prevent tumor progression by secreting immunoglobulins (Igs), promoting the T-cell response, presenting antigens, and killing cancer cells directly^13^. In contrast, regulatory B cells increase tumor activity through immunosuppressive factors, such as IL-10 and TGF-β^14,15^. In mice with melanoma and no B cells, subcutaneous tumor growth decreased or was eliminated. These effects were associated with enhanced effector function of T and/or natural killer cells negatively regulated by B cells^16^.

Specific subsets of B cells have been identified in the TME. TET2 is predominantly an epigenetic regulatory enzyme in B cells. Its activation is triggered by oxidative stress from the HCC TME and promoted by IL-10 expression. TET2 inhibition in B cells decelerates HCC progression by preventing the generation of IL-10^+^ B cells, thereby enhancing the antitumor response of CD8^+^ T cells and facilitating antitumor immunity to improve anti-PD-1 therapy for HCC^17^. Most B cells in the TME were positive for IgA. Further, IgA^+^ B cells expressed high immune regulatory molecules, including PD-L1, IL-10, and TGF-β.

In colorectal cancer, IgA^+^ B cells repressed the proliferation and activation of CD8^+^ T cells. These activities were regulated by miR15A and miR16-1, inactivated the NF-κB and STAT1 pathways, and reduced the production of chemokines CXC9 and CXC10^14^. CD27^+^CD21^+^ B cells are potential therapeutic targets in NSCLC immunotherapy because of their association with tumor progression and enhanced expression of the Tregs effector T-cell response^18^.

FCER2, the CD23 molecule, is an Fc receptor specific for IgE (Fcε RII) expressed on the surface of B and dendritic cells in mice and B cells and various other hematopoietic cells in humans. FCER2 expression negatively regulates B-cell receptor (BCR) by promoting actin-mediated BCR central cluster formation and B-cell activation. Colligation of FCER2 with the BCR reduces proliferation and increases B-cell apoptosis^19^. A lack of FCER2 enhanced B-cell spreading, concurrent with accumulated actin and increased levels of phosphorylated tyrosine, Btk, and F-actin and its downstream promoting regulator Wiskott Aldrich syndrome protein (WASP)^20^. Our in-silicon and *in vitro* experiments confirmed that FCER2 acted as a tumor-suppressing factor. FCER2^+^ B cells might inhibit tumor proliferation and migration. Our findings require replication in future, well-powered studies to further validate the mechanistic role of FCER2.

In contrast to B cells, decreased enrichment of macrophages was found in HPV + HNSCC and was associated with a worse prognosis. Recent work has highlighted the importance of tumor-associated macrophages in exacerbating desmoplasia, angiogenesis, nutrient deprivation, and immune suppression to promote tumor growth and regulate therapy resistance. Macrophages are mainly classified into pro-inflammatory M1 and immune-tolerant M2 (M2a, M2b, M2c, and M2d)^21,22^. The M1-polarized macrophages secrete TNF-α, IL-1, IL-6, IL-12, IL-23, CCL8, monocyte chemotactic protein-1 and 2 (MCP-1, MIP-2), ROS, cyclooxygenase-2 (COX-2), CD16 and CD32, and inducible NO synthase (iNOS). The M2-polarized macrophages produce high levels of IL-1 receptor antagonist (IL-1RA), arginase 1 (Arg-1), IL-10, TGF-β, CCL18, and low levels of IL-12^23^. However, subpopulations of macrophages and phenotypes found with HNSCC may be more heterogeneous. Definitions of M1 and M2 do not accurately describe this disease’s heterogeneity, which requires (and would be fully reflected by) further single-cell analyses.

We also conducted a bulk-cell–based TF network analysis of the TME in HNSCC. We identified 50 TFs that regulate B cells and 21 TFs that regulate plasma cells. Of these, SPI1 was a key regulator of the immune system signaling. Sixty-one percent of SPI1 target genes encode proteins that function in pathways, including antibodies and antibody receptors, and cytokines and cytokine receptors that regulate leukocyte development and inflammation. SPI1 defects lead to defects in B cells and other granulocytes, monocytes, and macrophages^24^. TAF is a checkpoint regulator of many TFs. TAF7 binds to and inhibits the kinase activity of TFIIH, BRD4, and P-TEFb, preventing them from functioning prematurely. TAF7 is essential for the differentiation and proliferation of immature thymocytes. Mouse embryonic fibroblasts with TAF7 knockdown demonstrate disabled transcription and stop proliferating. In T cells, TAF7 deletion affects several transcripts and inhibits activation and expansion in response to antigenic stimuli^25,26^. Thus, the TME appears to serve a regulatory function within the HNSCC. This issue deserves further exploration.

In conclusion, patients with HPV+ HNSCC demonstrate a TME that differs from those with HPV− tumors. We observed and focused on increased B and plasma cells in HPV+ tumors. These features were associated with improved survival. In contrast, HPV− tumors had significant increases in macrophages. We also found FCER2 to be a potential tumor-suppressing B-cell surface marker. FCER2 + B cells inhibit tumor proliferation and migration *in vitro*. Our results will provide an opportunity to better understand the TME of HNSCC and help tailor HPV+ HNSCC treatments to individual patients, and may serve as a reference dataset and resource for future in-depth exploration. This analysis patterns could be also applied to studies using other such data or of other tumor types by standard preprocessing and statistical analyses^12^. Also, further and more detailed investigations are needed to confirm our findings.

## Methods

### Data sources and acquisition

Single-cell RNA sequencing (scRNA-seq) datasets were downloaded from the Gene Expression Omnibus through accession number GEO: GSE139324 (https://www.ncbi.nlm.nih.gov/geo/)^27^, which was obtained from 10× technology in the form of RSEM normalized counts. The study included measurements from 131,224 individual immune cells in the TME from patients with HPV− (n = 18) and HPV + HNSCC (n = 8) who were immunotherapy treatment-naïve. Bulk RNA-seq and clinical data of HNSCC samples with recorded HPV status were obtained from Genomic Data Commons Data Portal (https://portal.gdc.cancer.gov/) based on the ‘hpv_status_by_ish_testing’ filter, which contained 19 HPV+ and 64 HPV- samples.

### Processing of scRNA-seq and bulk RNA-seq data

Quality filtering of the raw scRNA-seq data was performed using the DropletUtils package and R/Bioconductor package “skater”^28^. According to the median number of genes, cells with >10% of mitochondria-expressed genes and <10% of ribosome-expressed genes were filtered. After data normalization, the 2000 most-variable genes in single cells were identified using the Seurat package^29^. A principal component analysis was performed using variable genes as the input. Initial principal components were identified using the FindNeighbors and FindClusters functions of the Seurat package. Then, the identified clusters were reduced dimensionality using the uniform manifold approximation and projection (UMAP) method. For subclustering analysis, differentially expressed marker genes for each cell type (log_2_FC ⩾ 0.1, logFC > 0.25) were found using the FindMarkers function. Cell subclusters were annotated based on the obtained top logFC 500 marker genes. Cell types and cell type-specific gene expressions were calculated using the CIBERSORTx and CIBERSORT analytical web frameworks^30,31^. Cytokine genes were obtained using the AUCell R package. The AUC estimates the proportion of genes in the gene set that are highly expressed in each cell. According to the calculated AUC value, gene-expressing rankings were built for each cell, and the relative activity was shown^10^. The GO and KEGG enrichment analysis (https://www.kegg.jp/kegg/kegg1.html) used Clusterprofiler v3.16.1. TFs and cell states were identified using the Single-Cell Regulatory Network Inference and Clustering method^32^. Kaplan–Meier curves were performed using a survival package according to the established risk score and clinical data^33^.

### Patient samples and histopathologic staining

Patients undergoing surgical resection of HNSCC at our institution were considered candidates for enrollment. Informed consent was obtained from all patients. The study was conducted according to the principles expressed in the Declaration of Helsinki, and prior approval was obtained from the Medical Ethics Committee. Formalin-fixed, paraffin-embedded tissue samples were obtained at surgery and processed using routine hematoxylin and eosin staining, immunohistochemistry, and immunofluorescence staining. Antibodies used in this study included CDKN2A/p16INK4a (#BF0580), FCER2 (#DF6650) and CD19 (#DF7030, Affinity Biosciences, Jiangsu, China).

### Flow cytometry

Flow cytometry was used to assess the following cell lineages: B cells (CD3^−^CD19^+^), plasma cells (CD3^−^CD138^+^), FCER2^+^ B cells, and FCER2^−^ B cells. PBMCs from the above patients were isolated from whole blood using density gradient centrifugation in Ficoll/Hypaque for 20 minutes at 400 × g with the brake-off. The PBMCs were washed and stained with the indicated fluorescence-labeled antihuman antibodies for 30 min on ice and protected from light. The cells were then washed and fixed in 2% formaldehyde. The samples were acquired using a FACSCanto cytometer and analyzed using FlowJo v.10 (Treestar, Seattle, California, USA). The corresponding isotype controls were used to determine the negative gate. Antibodies used in this study included CD23-FITC (#338505), CD19-PE (#302207), CD3-APC (#300311), and CD138-APC-Fire^TM^ 750 (#352315, BioLegend, San Diego, California, USA).

### Cell proliferation and migration assay

The HPV+ HNSCC cell line SCC090 cells^34^ (RRID: CVCL_1899; ATCC, Manassas, VA, USA) and cultured routinely in Dulbecco’s Modified Eagle Medium (DMEM) supplemented with 10% fetal bovine serum (FBS) at 37 °C in 5% carbon dioxide. All experiments were performed with mycoplasma-free cells. The cell line was authenticated using STR profiling within the last 3 years. The PBMCs of the patients mentioned above were enriched for B cells by negative selection using the Human Pan B-cell isolation kit (Miltenyi Biotech, GmbH -Bergisch Gladbach, Germany) and sort purified by FCER2. SCC090 cells and indicated B cells were cocultured in DMEM without FBS. After 36 h of coculture, the supernatant was collected and used for further assay. Scratch wound, transwell migration, and CCK-8 proliferation assays were performed as previously described^35^.

### Statistical analyses

The results of *in vitro* experiments are expressed as the mean ± SEM and processed using SPSS statistical software, version 18.0 (IBM, Armonk, NY). The Student *t*- or Mann-Whitney U-tests were applied to determine between-group statistical differences. Differences of p < 0.05 were considered statistically significant.

### Supplementary information

Supplementary figures and table

## Data Availability

The data that support the findings of this study could be achieved from Gene Expression Omnibus (https://www.ncbi.nlm.nih.gov/geo/query/acc.cgi) with the accession number GSE139324^27^, and Genomic Data Commons Data Portal (https://portal.gdc.cancer.gov/, and the list of file identifiers is available on figshare (10.6084/m9.figshare.22292479)^36^. The analysis results associated with this paper is available on figshare (10.6084/m9.figshare.22292479)^36^.

## Code availability

The R code used in the analysis of the data is available on figshare (10.6084/m9.figshare.22292479).
