# Supplementary material for: Characterization of immune microenvironment in patients with HPV-positive and negative head and neck cancer
Source: Sci Data. 2023 Oct 12;10:694. doi: 10.1038/s41597-023-02611-3 (PMC10570276; doi:10.1038/s41597-023-02611-3)
Supplement: Supplementary file 1 — Supplementary figures and table [file 41597_2023_2611_MOESM1_ESM.docx]

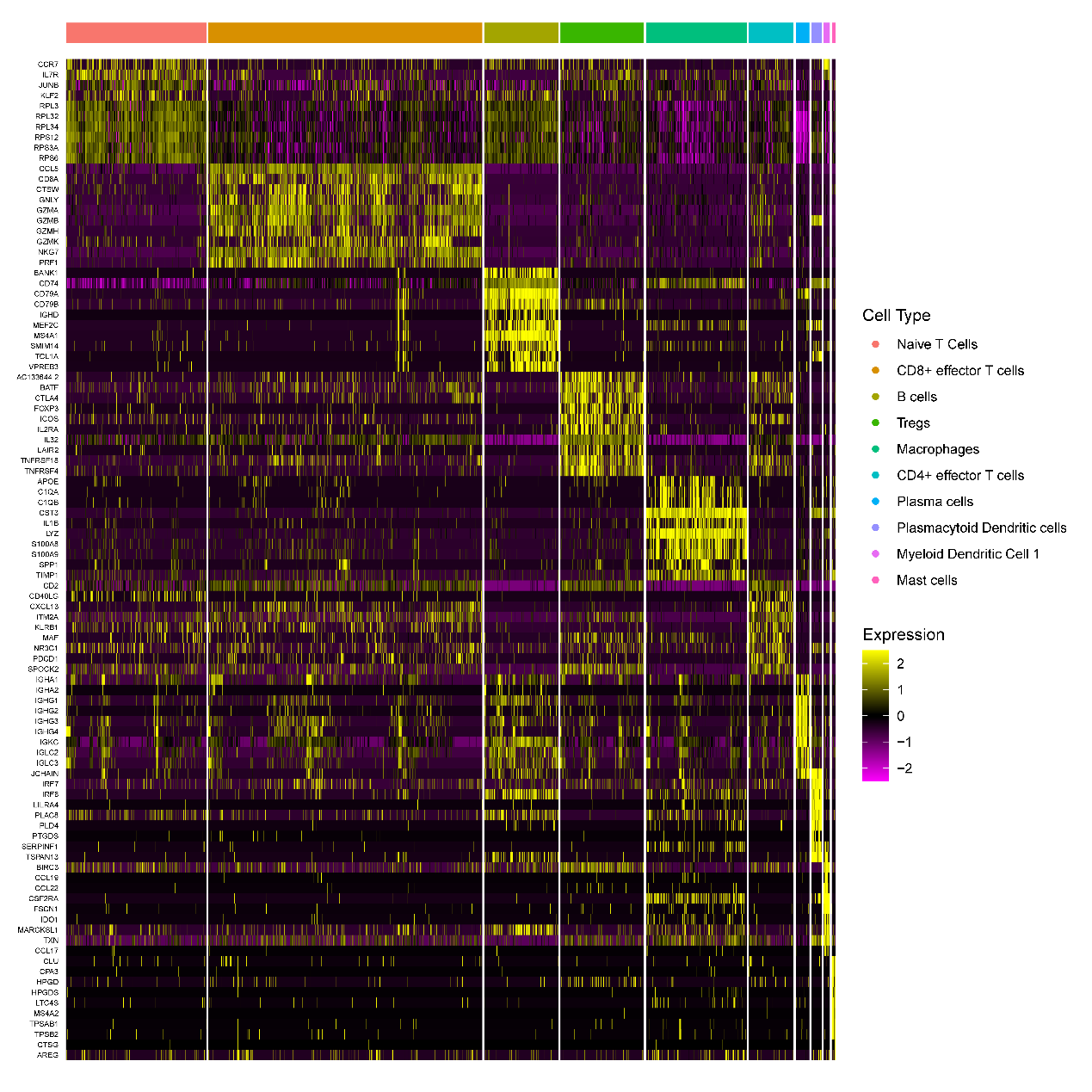


Figure S1. Heatmap showing expression of the top 10 new marker genes in each cell type.


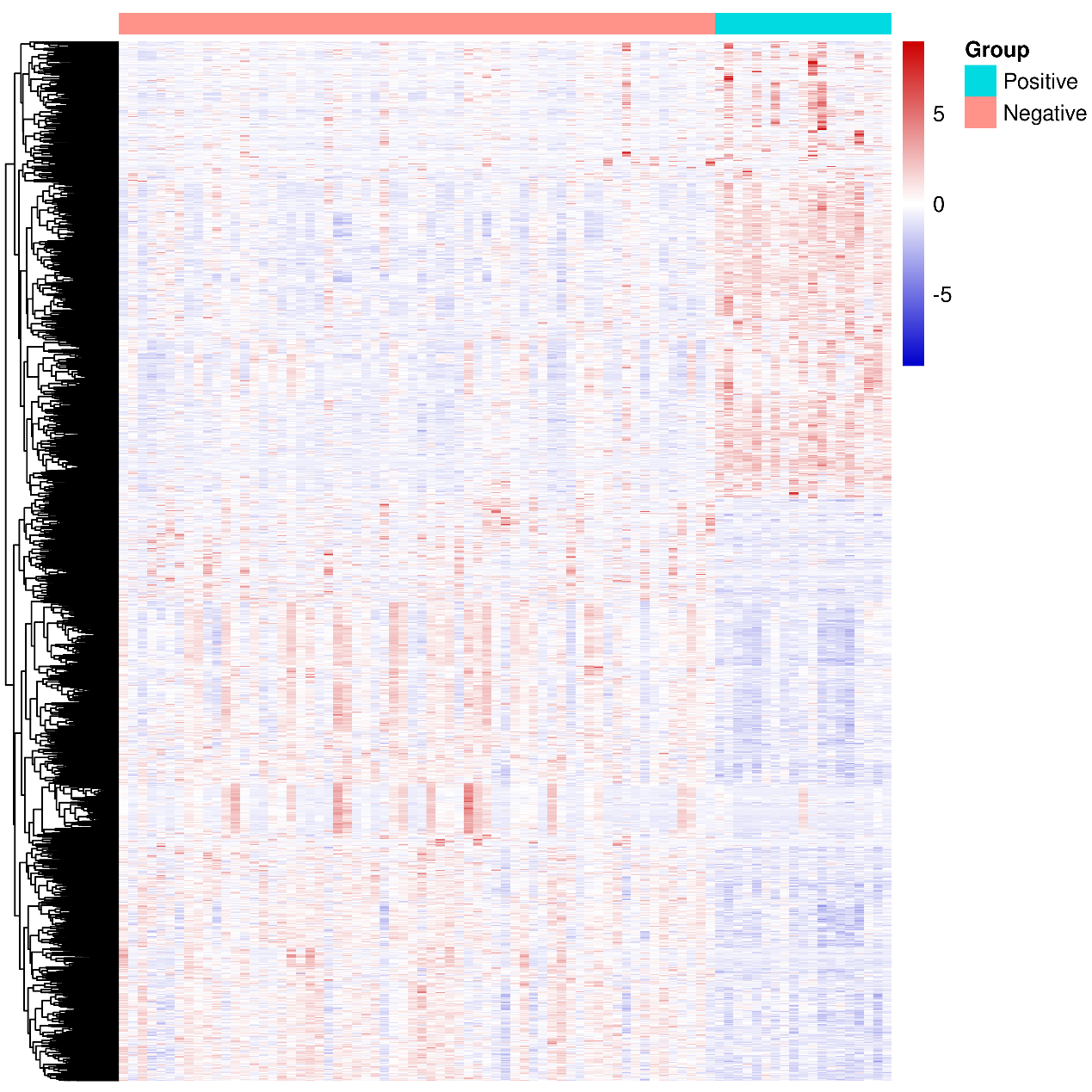


Figure S2. Heatmap showing differential gene expression in HPV+ and HPV- tumors.


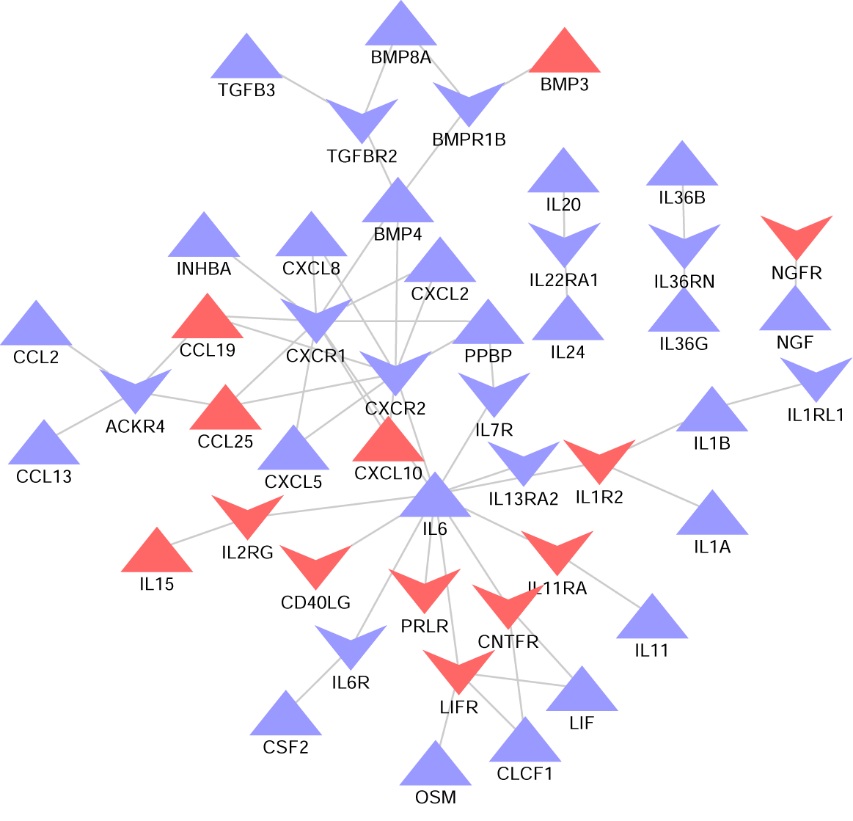


Figure S3. Cytokine-receptor interaction network of differential expressed genes from the KEGG analysis. Red color, up regulated genes; Purple color, down regulated genes; triangle represent ligand, and V represent receptor.

Table S1. Transcription factors related to cytokine regulation revealed by bulk-cell analysis.

| TF | Gene |  | TF | Gene |  | TF | Gene |
| --- | --- | --- | --- | --- | --- | --- | --- |
| CEBPB | OSM |  | STAT3 | CD40LG |  | TFAP2C | LIF |
| CTCF | CXCL2 |  | STAT3 | IL15 |  | SPI1 | IL6 |
| EGR1 | LIF |  | STAT3 | IL1B |  | SPI1 | INHBA |
| EGR1 | OSM |  | STAT3 | IL1R2 |  | JUN | FASLG |
| EP300 | IL1R2 |  | STAT3 | IL1RL1 |  | NOTCH1 | CD27 |
| EP300 | LIF |  | STAT3 | IL21R |  | SMAD3 | LIF |
| FOXA2 | CXCL2 |  | STAT3 | IL6 |  | TFAP2A | CCL2 |
| GATA1 | CXCL8 |  | STAT3 | IL7R |  | RUNX2 | CXCL2 |
| GATA2 | CXCL2 |  | STAT3 | INHBA |  | TP63 | OSM |
| IRF1 | IL15 |  | STAT3 | LIF |  | SPI1 | LTB |
| JUN | CCL2 |  | STAT3 | OSM |  | TP63 | LIF |
| JUN | IL21R |  | STAT3 | TNFRSF17 |  | SPI1 | CXCL2 |
| JUN | IL6 |  | MYC | XCL2 |  | SRF | IL6 |
| KDM5B | LIF |  | NANOG | TNFRSF13C |  | SPI1 | CCL2 |
| MYC | IL6 |  | SUZ12 | INHBA |  | ARID3A | CXCL2 |
| MYC | TTNFRSF13C |  | NR3C1 | CXCL2 |  | ATF1 | CXCL2 |
| RAD21 | CXCL2 |  | YY1 | IL6 |  | ATF3 | TNFRSF13C |
| RAD21 | IL6 |  | RCOR1 | LIF |  | BACH1 | IL6 |
| SPI1 | CXCL10 |  | RELA | IL15 |  | BCL3 | CXCL2 |
| SPI1 | OSM |  | SPI1 | IL7R |  | BHLHE40 | CXCL2 |
| TCF3 | IL2RG |  | REST | IL2RG |  | BHLHE40 | IL2RG |
| TCF3 | OSM |  | REST | LIF |  | BHLHE40 | LIF |
| ATF3 | IL6 |  | REST | TNFRSF17 |  | BHLHE40 | OSM |
| CEBPB | FASLG |  | STAT3 | CXCL2 |  | BHLHE40 | TNFRSF13C |
| CUX1 | CXCL2 |  | STAT3 | IFNG |  | CBX3 | CXCL2 |
| CUX1 | IL1RL1 |  | TRIM28 | IL6 |  | CEBPB | CD27 |
| CUX1 | IL21R |  | SETDB1 | IL18 |  | CEBPB | CXCL2 |
| EGR1 | FASLG |  | STAT5A | IL15 |  | CEBPB | CXCL8 |
| EGR1 | IL15 |  | TAL1 | IL1R2 |  | CEBPB | IL18 |
| EGR1 | TNFRSF13B |  | SPI1 | IL1B |  | CEBPB | IL1B |
| ELK1 | IL15 |  | SPI1 | IL1R2 |  | CEBPB | IL1RL1 |
| EP300 | TNFRSF13B |  | AR | CXCL8 |  | CEBPB | IL21R |
| ESR1 | IL1R2 |  | AR | CXCL2 |  | CEBPB | IL6 |
| MYC | IL21R |  | FLI1 | CXCL8 |  | CEBPB | IL7R |
| MYC | TNFRSF17 |  | ARNT | IL6 |  | CEBPB | INHBA |
| FOXP2 | LIF |  | ATF3 | OSM |  | CEBPB | TNFRSF13B |
| GATA1 | IL1R2 |  | ATF3 | IL7R |  | CEBPD | CXCL2 |
| GATA1 | IL1RL1 |  | ATF3 | INHBA |  | CHD1 | LIF |
| GATA1 | LIF |  | MECOM | OSM |  | CHD1 | OSM |
| GATA2 | CXCL8 |  | CEBPB | CCL2 |  | CREB1 | CXCL2 |
| GATA2 | IL1B |  | FLI1 | CD27 |  | CREB1 | IL21R |
| GATA2 | IL1RL1 |  | FLI1 | CCL19 |  | CREB1 | IL2RG |
| GATA2 | IL6 |  | ERG | IL21R |  | CREB1 | OSM |
| TAL1 | IL6 |  | ERG | CXCL2 |  | CREB1 | TNFRSF13C |
| HNF4A | CXCL2 |  | ERG | CCL2 |  | CTBP2 | LIF |
| HNF4A | LIF |  | KLF4 | IL15 |  | CTCF | CCL19 |
| RAD21 | TNFRSF13C |  | MECOM | CXCL2 |  | CTCF | IL18 |
| RELA | CXCL10 |  | FLI1 | IL1B |  | CTCF | IL1R2 |
| REST | CCL2 |  | MYB | OSM |  | CTCF | IL21R |
| SETDB1 | IL1B |  | FLI1 | IL21R |  | CTCF | IL6 |
| CTCF | LTA |  | EP300 | IL7R |  | MAZ | CXCL2 |
| CTCF | LTB |  | EP300 | LTA |  | MAZ | IL15 |
| CTCF | OSM |  | EP300 | LTB |  | MAZ | IL21R |
| CTCF | TNFRSF13C |  | EP300 | OSM |  | MAZ | IL2RG |
| CTCF | TNFRSF17 |  | ETS1 | CD27 |  | MAZ | IL6 |
| CTCFL | TNFRSF13C |  | ETS1 | CXCL2 |  | MAZ | INHBA |
| E2F1 | CXCL2 |  | ETS1 | IL15 |  | MAZ | LIF |
| E2F6 | CXCL2 |  | ETS1 | IL21R |  | MAZ | LTA |
| E2F6 | IL6 |  | ETS1 | IL2RG |  | POLR2A | TNFRSF13C |
| E2F6 | LIF |  | ETS1 | IL6 |  | RAD21 | IL18 |
| E2F6 | OSM |  | ETS1 | LTA |  | RBBP5 | CXCL2 |
| E2F6 | TNFRSF13C |  | ETS1 | LTB |  | RBBP5 | TNFRSF13C |
| EBF1 | CD27 |  | ETS1 | OSM |  | RUNX3 | CD27 |
| EBF1 | IL1R2 |  | FLI1 | IL2RG |  | RUNX3 | TNFRSF13C |
| EBF1 | IL21R |  | FOS | IL6 |  | SAP30 | LIF |
| EBF1 | IL6 |  | FOSL1 | CXCL8 |  | SIN3A | LIF |
| EBF1 | LIF |  | HDAC1 | CXCL2 |  | SMARCB1 | CXCL2 |
| EBF1 | OSM |  | HDAC1 | IL1B |  | SMC3 | IL18 |
| EBF1 | TNFRSF13C |  | HDAC1 | IL2RG |  | SMC3 | IL6 |
| EBF1 | TNFRSF17 |  | HDAC1 | LIF |  | SMC3 | INHBA |
| EGR1 | IL2RG |  | HDAC1 | OSM |  | SP1 | TNFRSF13C |
| EGR1 | TNFRSF13C |  | HDAC2 | CCL19 |  | ZBTB7A | IL2RG |
| ELF1 | CD27 |  | HDAC2 | CXCL2 |  | ZBTB7A | LIF |
| ELF1 | LIF |  | HDAC2 | CXCL8 |  | ZBTB7A | TNFRSF13C |
| ELF1 | OSM |  | HDAC2 | IL1B |  | ZNF263 | IL6 |
| EP300 | CCL2 |  | HDAC2 | IL1R2 |  | ZNF263 | LIF |
| EP300 | CD27 |  | HDAC2 | IL2RG |  | ZNF263 | TNFRSF13C |
| EP300 | CXCL2 |  | HDAC2 | LIF |  | ZNF384 | CD27 |
| EP300 | CXCL8 |  | IRF1 | CXCL10 |  | ZNF384 | IL6 |
| EP300 | IL15 |  | MAX | IL6 |  | ZNF384 | LTA |
| EP300 | IL18 |  | MAX | LIF |  | ZNF384 | LTB |
| EP300 | IL1B |  | MAX | OSM |  | ZNF384 | TNFRSF13C |
| EP300 | IL21R |  | MAX | TNFRSF13C |  |  |  |
| EP300 | IL6 |  | MAZ | CD27 |  |  |  |

TF, Transcription factor.
